# A Case Report of Pulmonary Embolism Caused by Substernal Goiter

**DOI:** 10.7759/cureus.26581

**Published:** 2022-07-05

**Authors:** Hongli Liu, Ming Chow

**Affiliations:** 1 Internal Medicine, Rochester Regional Health, Rochester, USA; 2 Pulmonary and Critical Care Medicine, Rochester Regional Health, Rochester, USA

**Keywords:** multinodular goiter, thromboembolic disease, anterior mediastinal mass, pulmonary embolism (pe), substernal goiter

## Abstract

Substernal goiter is overall an uncommon disease. Obstructive symptoms can occasionally develop in older patients with a longstanding history of goiter. Here, we describe a rare case of pulmonary embolism presenting as a complication of benign substernal goiter in a young patient without preceding recognized thyroid disease. After three separate biopsies, surgical resection was eventually performed, with pathology confirming the diagnosis of multinodular thyroid with cystic changes. Four months after the surgery, a CT angiogram of the chest was performed, which showed resolution of bilateral pulmonary emboli.

## Introduction

The term “goiter” refers to abnormal growth of the thyroid gland. In healthy adults without iodine deficiency, the mean sonographic thyroid volume is around 8 ml [1]. The American Thyroid Association defines a substernal goiter as a thyroid extending past the sternal notch with the patient in the supine position, detected either radiologically or clinically [2]. Enlargement of a substernal goiter can cause various symptoms from obstruction of structures in the thoracic inlet, most commonly dysphagia, dyspnea, and voice changes [3]. Here, we present a case of pulmonary embolism as a result of compression of the subclavian vein from a substernal goiter.

## Case presentation

A 35-year-old female with no significant past medical history presented to the hospital with worsening right anterior neck pain and swelling over two weeks. She reported no dyspnea or dysphagia. She denied any weight loss or night sweats. Her family history was notable for benign thyroid disease causing esophageal compression in her mother.

**Figure 1:**
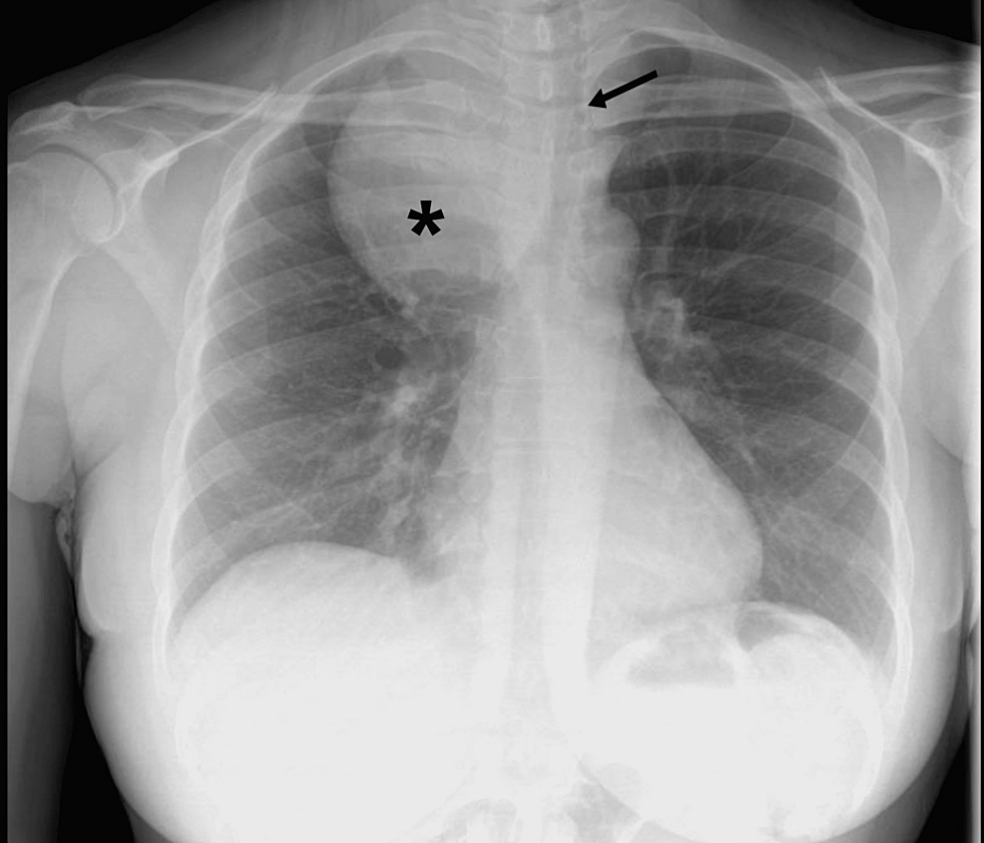
Chest X-ray Chest X-ray showing a right upper lung zone mass (asterisk) and leftward tracheal deviation (arrow).

On presentation, she was hemodynamically stable, saturating well on room air, and not in respiratory distress. On physical examination, a firm, non-mobile, non-tender mass was palpated medial to the right sternocleidomastoid muscle and superior to the supraclavicular fossa. The left neck appeared full. Routine blood work revealed a normal complete blood count (CBC), basic metabolic panel (BMP), and a mildly depressed thyroid-stimulating hormone (TSH) of 0.43 uIU/ml (normal range: 0.55-4.78 uIU/ml) with normal free T4. Chest X-ray (Figure 1) showed right upper lung zone mass with leftward tracheal deviation.

**Figure 2:**
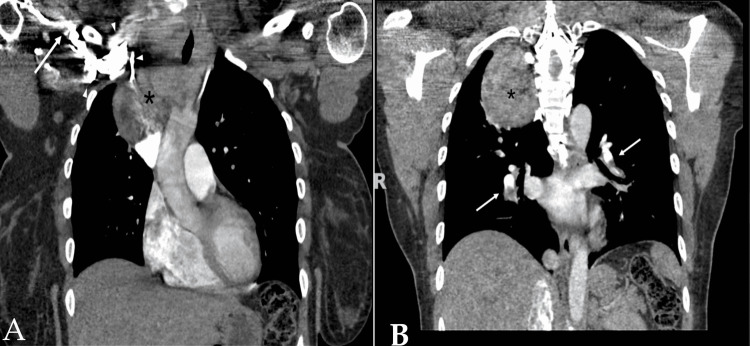
Coronal CT of the chest with IV contrast (A) Heterogenous thyroid mass extending into the right thoracic cavity (asterisks). Subclavian vein (arrow) can be seen with collateral vessels (arrowhead). (B) Bilateral pulmonary embolism (white arrows).

CT of the neck and chest showed diffusely enlarged and heterogeneous thyroid mass extending into the right thoracic cavity, with intrathoracic component measuring 7.7 x 7.0 x 9.6 cm in the anteroposterior, transverse, and caudocranial dimensions, respectively. There was near-complete occlusion of the right subclavian vein (Figure 2A) and multiple bilateral pulmonary emboli (Figure 2B).

She was started on therapeutic enoxaparin for anticoagulation. Bilateral lower extremity venous ultrasound showed no evidence of deep venous thrombosis. Ultrasound-guided fine-needle biopsy of thyroid revealed follicular cells in a colloid background. Due to concerns that malignancies may not be adequately evaluated with fine needle biopsy, a CT-guided core biopsy of the upper mediastinal part of the lesion was subsequently performed. Pathology showed benign thyroid tissue consistent with nodular hyperplasia. She was discharged with enoxaparin and planned for readmission for surgery. To further exclude malignancy, including primary thyroid lymphoma in this heterogeneous mass, she underwent an outpatient endobronchial ultrasound (EBUS) guided fine needle aspiration of the lower mediastinal part of the lesion. Results were consistent with benign thyroid tissue. Following this, robotic video-assisted right thoracoscopy and thyroidectomy were performed by ENT and cardiothoracic surgery. Surgical pathology revealed multinodular thyroid with cystic changes and was negative for malignancy.

At four months follow-up, her symptoms had resolved, and she exhibited no signs of hypothyroidism. Repeat CT angiogram of the chest showed resolution of pulmonary embolism. As she had no previous personal history or family history of venous thromboembolic disease and had an apparent anatomic reason for this event, she was considered to have a provoked pulmonary embolism, and thrombophilia testing was not recommended. After six months of full anticoagulation, she was continued on rivaroxaban 10 mg daily for thromboprophylaxis until 12 months after surgery.

## Discussion

Large substernal goiter is overall an uncommon disease. Analysis of the National Inpatient Sample indicates that substernal goiter represented 5% of 110,889 total thyroidectomies performed between 2000 and 2010 [4]. At the time of surgery, the average age was 52 years, with female predominance (76%). Compressive symptoms can be caused by progressive growth and narrowing of the thoracic outlet or sudden enlargement due to hemorrhage into a nodule. In a case series, 70% of substernal goiter patients have a preceding cervical mass for years before developing compressive symptoms [5]. In contrast to the typical scenario, our patient presented with compressive symptoms at a young age (35 years old) without any preceding thyroid disease.

Venous thromboembolism (VTE) is a recognized complication of hyperthyroidism. On the contrary, pulmonary embolism has rarely been described in patients with benign substernal goiter without hyperthyroidism [6,7]. In both reported cases, patients were elderly (75 and 68 years old, respectively) and had a longstanding history of goiter (40 years and 20 years, respectively). In our case, pulmonary embolism was postulated to be caused by compression of the right subclavian vein, although the initial CT study was limited due to contrast timing. Despite the fact that her TSH was slightly depressed, free T4 was within normal limits, and there was no prior history of hyperthyroidism.

According to a systematic review, the most common causes of anterior mediastinal mass in adults are thymic malignancy (approximately 35%), lymphoma (about 25%), thyroid and other endocrine tumors (approximately 15%), benign teratoma (approximately 10%), and malignant germ cell tumors (approximately 10%) [8]. The decision to biopsy should be individualized. Confirming the diagnosis with biopsy is preferred when lymphoma is suspected, given that lymphoma usually can be treated with a non-surgical approach. Standard biopsy techniques include percutaneous, endobronchial, or surgical methods. In a single-center review, the diagnostic yield of CT-guided percutaneous biopsy of an anterior mediastinal mass is around 77%. Lymphoma is the most common cause of non-diagnostic results [9]. Even though none of the core biopsies was discordant with surgical pathology in the aforementioned study, in this case, thyroid nodular hyperplasia from the CT-guided core biopsy is a common pathology and may represent incidental benign findings. Thus, it is prudent to rule out lymphoma given her age. While the collateral vessels (as seen in Figure 2A) attest to the chronicity of the disease process, due to the rareness of this condition at her age, she underwent three separate biopsies to exclude underlying malignancies before surgery.

## Conclusions

Pulmonary embolism is a rare complication of benign substernal goiter due to compression of intrathoracic vasculature. Malignancy, including lymphoma, needs to be excluded in young patients presenting with an anterior mediastinal mass. After confirmation with biopsy, surgery for substernal goiter can be successfully performed without the need for sternotomy.
